# Deep Ontology Alignment Using a Natural Language Processing Approach for Automatic M2M Translation in IIoT

**DOI:** 10.3390/s23208427

**Published:** 2023-10-12

**Authors:** Saleha Javed, Muhammad Usman, Fredrik Sandin, Marcus Liwicki, Hamam Mokayed

**Affiliations:** 1 Machine Learning, Department of Computer Science, Electrical and Space Engineering, Lulea University of Technology, 97187 Lulea, Swedenmarcus.liwicki@ltu.se (M.L.);; 2 Department of Computer Science, National University of Computer and Emerging Sciences, Chiniot-Faisalabad Campus, Chiniot 35400, Pakistan

**Keywords:** ontology alignment, M2M translation, self-attention, deep learning, Industry 4.0, Industry 5.0 IIoT, knowledge graph, industrial internet of things, smart city

## Abstract

The technical capabilities of modern Industry 4.0 and Industry 5.0 are vast and growing exponentially daily. The present-day Industrial Internet of Things (IIoT) combines manifold underlying technologies that require real-time interconnection and communication among heterogeneous devices. Smart cities are established with sophisticated designs and control of seamless machine-to-machine (M2M) communication, to optimize resources, costs, performance, and energy distributions. All the sensory devices within a building interact to maintain a sustainable climate for residents and intuitively optimize the energy distribution to optimize energy production. However, this encompasses quite a few challenges for devices that lack a compatible and interoperable design. The conventional solutions are restricted to limited domains or rely on engineers designing and deploying translators for each pair of ontologies. This is a costly process in terms of engineering effort and computational resources. An issue persists that a new device with a different ontology must be integrated into an existing IoT network. We propose a self-learning model that can determine the taxonomy of devices given their ontological meta-data and structural information. The model finds matches between two distinct ontologies using a natural language processing (NLP) approach to learn linguistic contexts. Then, by visualizing the ontological network as a knowledge graph, it is possible to learn the structure of the meta-data and understand the device’s message formulation. Finally, the model can align entities of ontological graphs that are similar in context and structure.Furthermore, the model performs dynamic M2M translation without requiring extra engineering or hardware resources.

## 1. Introduction

The speed of technological development is changing with automation and digitization, bringing several challenges [1]. The backbone of Industry 4.0 and 5.0 was industrial automation systems that enabled sustainable development [1] and gave innovative functionalities access to the cyber world [2], known as cyber-physical systems (CPS). CPS is a conjunction between physical systems and digital micro-systems that features a tight integration of modeling, computation, and communication. Cyber-physical systems and the IoT have begun merging in the industrial digitization process, further known as the industrial internet of things (IIoT). The focus of such mergers has been reshaping society [2] by bridging physical divides via digital connectivity using IIoT and digitization applications. Applications include automation of manufacturing processes [3,4], agriculture for precision fertilization programs [5], smart farming, condition monitoring of wind turbines [6] and farms, smart factories [7], smart buildings and cities [8], and many others. By digitizing physical processes, these applications have lowered the overheads associated with human dependency, as well as the cost, time, and computation required. While these solutions aim to achieve connectivity across their respective service-oriented architectures (SOA), when it comes to developing a dynamically scalable and enhanced software-as-a-service (SaaS) architecture that can incorporate machine learning models as a service (MLaaS) [9], such systems are still in their infancy. Additionally, this problem becomes more challenging and crucial in the environmental settings of Industry 5.0. This application domain involves a hub of devices with different responsibilities working together for the same business objective. Despite these devices having homogeneous or heterogeneous underlying structures, the devices need to comprehend, translate, and interact with each other, to converge toward the business goal. Thus, IIoT automation cannot be confined to the digitization of connections, and this development is subject to interoperability challenges. In particular, machine learning (ML) approaches are considered, to automate costly engineering processes. For example, challenges related to the automatic translation of messages transmitted between heterogeneous devices are investigated using supervised and unsupervised machine learning approaches [10].

We conceive IIoT device ontology as a device’s language, corresponding to the language encoder component. The schema of the ontology graph contains all the information about classes and the sub-class hierarchy and their connections, which we convert into a structural encoder. Then, the names of classes and relations are considered labels mapped as side information in the ontology graph and as sentence tokens in the NLP paradigm. Finally, relations indicate which classes are interconnected, and these constitute a structural question set. To the best of our knowledge, no other work in the literature has proposed this mapping, and so there is a knowledge gap regarding the efficient use of such synergies. The existing techniques of entity alignment are based on different approaches for integrating structural information, which overlook that, even if a node pair have similar entity labels, they may not belong to the same ontological context, and vice versa. To address these challenges, a model based on modifying the BERT-interaction model on graph triples was developed. The developed model is an iterative model for the alignment of heterogeneous IIoT ontologies, enabling alignments within nodes and relations. When compared to the state-of-the-art BERT INT, on the DBPK15 language dataset, the developed model exceeded the baseline model by an error rate of 2.1%. This work can be considered a step towards enabling translation between heterogeneous IoT sensor devices; therefore, the proposed model could be extended to a translation module in which, based on the ontology graphs of a device, the model can interpret the messages transmitted from that device.

We focus on designing an ontology alignment model as a first step toward developing automatic dynamic translation between IIoT heterogeneous devices. The proposed model could be embedded into dynamic automated IIoT applications with multiple interconnected and heterogeneous devices, for IIoT applications that require intercommunication for performing a mutual task, such as condition monitoring of wind turbines [6] or access control systems [3]. Our model can utilize online M2M translation across devices with varying ontologies, to allow seamless operations. The following summarizes our main contributions:

- Thoroughly investigate how to enable automatic alignment across heterogeneous IIoT sensor devices using an NLP-based learning model, in conjunction with entity alignment for the ontology graph;
- Explore the use of an ontology graph as the main metric in a representation learning problem, for interpreting the metadata of sensory devices;
- The first significant novelty herein is highlighting three knowledge gaps: (1) the lack of research attention on modeling ontology alignment approaches for IIoT heterogeneous devices, (2) the scarcity of literature on fusing NLP methodologies with the IIoT domain, and limitations of datasets for IIoT ontology alignment;
- The second prime novelty of this work is synthesizing a model as a solution for the IIoT ontology alignment task. The model significantly exceeds the state-of-the-art results on the DBP15K languages dataset by a wide margin. This work is the first of many to conceptualize a mapping between NLP and IIoT domains by utilizing knowledge graph modeling for the device ontology.

This paper is outlined in eight sections: first, a brief background is given in Section 2.1 of the various domains used in constituting the proposed solution. Section 2 presents the important state-of-the-art works in each domain. Then, a detailed discussion on the highlighted knowledge gaps is given in Section 2.4. Section 3 elaborates on the problem formulation, followed by Section 4 with the complete architecture of the proposed solution, followed by a use-case explanation for the proposed system discussed in Section 5. Then, Section 6 states the used experimental setup and a proof of concept with results is presented in Section 7. Lastly, reflections and concluding remarks are discussed in Section 8.

## 2. Related Work

The work presented herein is primarily in the context of the industrial Internet of Things paradigm. We address the translation problem amongst heterogeneous sensory devices, with respect to the ontology followed when installing the network in a smart building. Here, all devices are interconnected to regulate and optimize energy consumption, such as temperature control (heating or cooling), humidity, or climate. Each subsection presents the important state-of-the-art works in the various domains that have contributed to hypothesizing the research question and its solution.

### 2.1. Background

Numerous models, with various strengths and weaknesses, have been established for cross-language translation, but none have been designed for the IIoT automatic ontology paradigm. This section outlines the different dimensions involved in synthesizing the proposed solution. The first dimension involves the IIoT ontology’s constitution and role from an industrial perspective. The next dimension addresses the importance of interoperability in the context of ontologies and the popularity of ML for modernizing Industry 4.0 applications and leading toward Industry 5.0 smart-society applications.

#### 2.1.1. Interoperability in the Context of IIoT Ontologies

**Figure 1:**
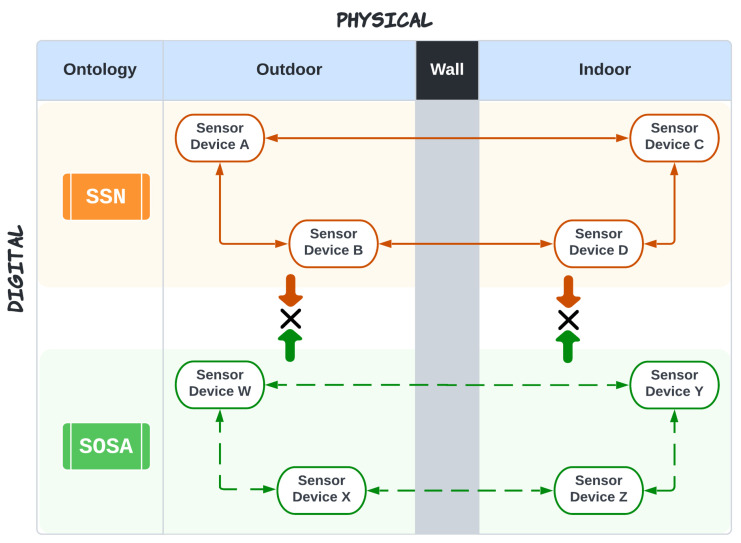
Explanation of heterogeneity in device ontology. The figure illustrates an example scenario of a smart building with multiple interconnected sensors installed outside and inside. Few devices follow the Semantic Sensor Network (SSN) ontology; the rest follow the Sensor-Observation-Sampling-Actuator (SOSA) ontology. All the devices that follow SSN ontology can intercommunication, and similarly, devices that follow SOSA ontology can successfully intercommunicate. However, a device following SSN ontology can not communicate with the device following SOSA [13].

With the development of embedded CPSs and vast computational resources, the IIoT has grown significantly, resulting in a massive increase in IoT devices. According to recent figures, the number of linked IoT devices globally reached 15.14 billion in 2023. This forecast is expected to quadruple to around 50 billion IoT devices by 2030 [11]. The IIoT is a hub for heterogeneous and homogeneous devices that require seamless integration and connectivity. The interoperability issue involves the challenge of enabling communication, despite varying assumptions about the data model, message format, and device ontology [12]. Figure 1 presents an example scenario of ontology interoperability. In the past decade, researchers have shown a keen interest in developing ML-based automatic translation models to solve interoperability problems, but a lack of datasets and the complexity constraints on real-world applications have hindered this synergy so far.

#### 2.1.2. Representation Learning for Sensor Devices

The performance of ML algorithms is highly dependent on the type of data representation used. As a result, a major percentage of the effort is spent on feature engineering to execute ML algorithms and build data transformations that result in a representation of the data suited for effective learning [14]. Data of sensor devices is conceptualized in several technical layers of SOAs. It includes the device’s ontology and protocol, data format, message payload schema, message transmission protocol, and more. However, this work emphasizes the importance of device ontology for identifying and disentangling the messages received from a heterogeneous device. Given any device’s messages and its ontology, the representation learning model can map vector representation of low-dimensional space for each entity in the ontology. The vectors of every unique entity are also unique, called embedding vectors. There are three major methods in which the model can perform representation learning: (1) supervised in which input labels and mapping of input X to output Y are given; (2) semi-supervised in which a mix of labeled data and unlabeled data are used; and (3) unsupervised in which no prior information of labels or mapping onto output is given. The present IoT sensor ontology domain literature has examples of supervised and semi-supervised approaches as discussed in Section 2 but lacks unsupervised learning-based methods.

#### 2.1.3. Sensor Ontologies

Sensors are a major source of data available on the Web today. While sensor data may be published as mere values, searching, reusing, integrating, and interpreting these data requires more than just the observation results. The captured information with its context is equally important for properly interpreting the values as information about the studied feature of interest; for example, for a heater, the observed property, the specific locations and times at which the temperature was measured, and a variety of other information. This work only takes into account the ontology that is standardized, integrated by, and aligned with W3C semantic web technologies [15] and linked data [16], which are key drivers for creating and maintaining a global and densely interconnected graph of data.

Intelligent sensors should be interconnected seamlessly and securely, to enable automated high-level smart applications. Smart interconnection of sensors, actuators, and devices enables the development of solutions required for smart city- and CPS industrial solutions [17].

**Figure 2:**
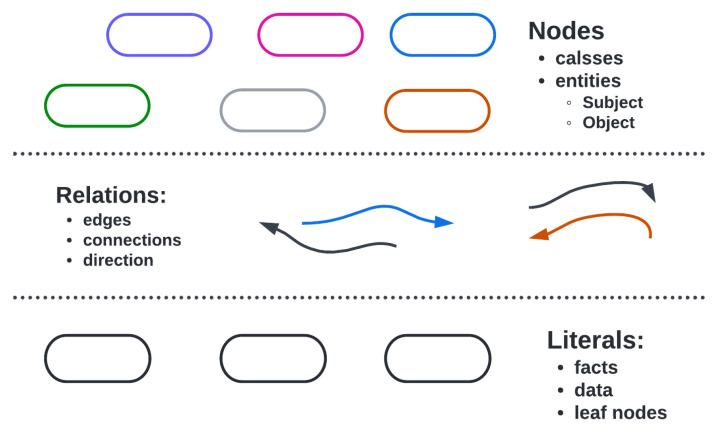
Basic Components of an Ontology. There are three types of nodes here: (1) subject node, (2) object node, and (3) literal node. Both subject and object nodes belong to the class of the knowledge domain, for which the ontology is developed. The edges between nodes represent relations, and the third literal node has a data fact about them.

Ontologies can enrich sensory data and ensure interoperability by providing an abstraction layer [18]. The ontology defines the semantic model and contextual information of the devices [19]. Figure 2 shows the essential components of an ontology design. W3C has developed several benchmark ontologies based on IoT standards, such as Smart Onto Sensor, SSN, SAN, IoT-Lite, SOSA, and others, adopted by industrial manufacturers globally. The authors of [18] presented a timeline of the evolution of all base-level ontologies developed from 2002 to 2018. The authors divided the timeline into before and after the SSN ontology, as this was the first ontology with complete design patterns for a sensory device network. Ontologies are continually evolving, compiling ever more space for reasoning and simplification.

**Figure 3:**
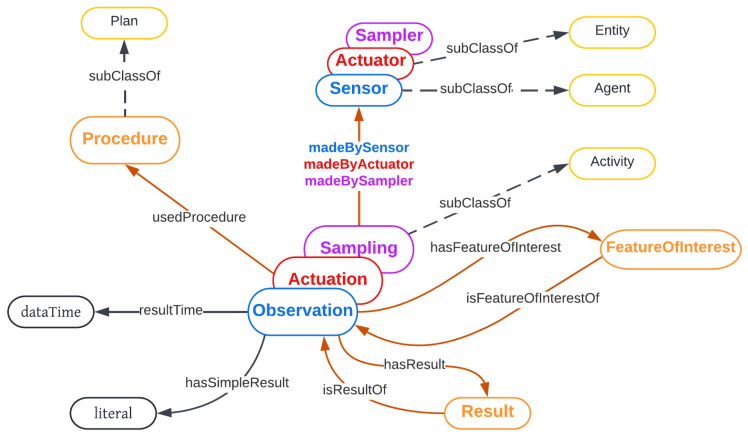
SSN and SOSA ontology core structure.

SOSA provides a lightweight core for SSN, as shown in Figure 3, and aims to broaden the target audience and application areas that can make use of semantic-web ontologies. At the same time, SOSA [20] acts as a minimal interoperability fall-back level; i.e., it defines the common classes and properties for which data can be safely exchanged across all uses of SSN [21], its modules, and SOSA.

### 2.2. M2M Translation Problem in the IIoT Domain

Devices often use different communication protocols, standards, and data representation languages, which creates interoperability and M2M translation challenges. The existing literature contains different perspectives on addressing the M2M translation problem. Application protocol-level solutions focus on predefined functions or annotations as proxies and XML schemes to enable translation between sender and receiver devices [22,23]. However, such solutions fail regarding automated CPSs, which cannot rely on hand-crafted predefined schemes for every possible pair of devices. Moreover, protocol-level proxies exclude the possibility of utilizing data in the messages to make intuitive interpretations about the device’s protocol. Data-driven methods [24] exploit the data augmentation approach to analyze patterns and features in device data messages and infer important knowledge that can generate interpretations between heterogeneous devices. However, to the best of our knowledge, a successful automatic translation model based specifically on industrial IoT ontologies has not been developed. The major challenge in developing such learning-dependent solutions is the unavailability of large datasets, which is a great hindrance.

### 2.3. Knowledge Graph Alignment

**Figure 4:**
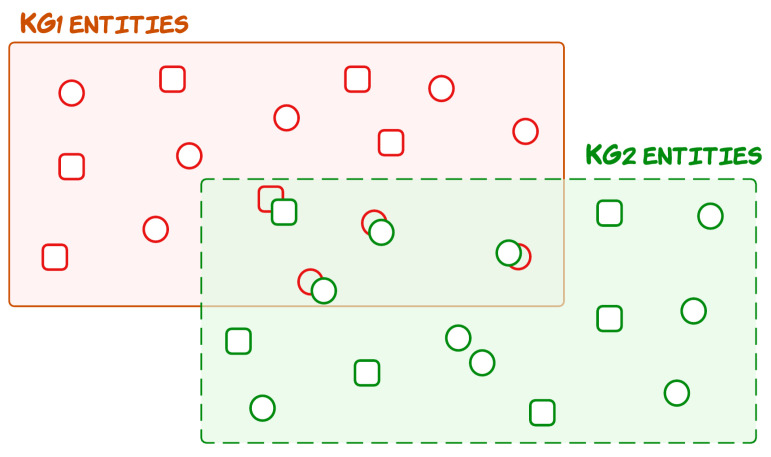
Illustration of entity alignment between two heterogeneous KGs. Each KG has its embedding vector space for its entities, i.e., circles represent nodes, and squares represent relations. The entities from both graphs that have similar embedding in the vector space overlap in the figure.

Knowledge graph alignment aims to link equivalent entities across different knowledge graphs. ML models, in conjunction with data-driven methods for automatic semantic translations, have recently been trending among researchers [10]. Deep learning (DL) models such as deep alignment for ontology [25] design solutions among parallel ontologies by aligning entities of different ontologies that have been developed independently but for the same domain. [25] introduced word vector-driven descriptions for defining the entities (nodes) and matching tasks on the DBpedia dataset for ontologies and Schema.org. Recently, a large number of knowledge graphs (KGs) have been established to support AI applications, such as Freebase [26] and YAGO [27]. Entity alignment seeks to discover identical entities in different KGs, such as the English entity Thailand and its French counterpart Thailande. To tackle this important problem, the literature has devised embedding-based entity alignment methods [28,29,30]. These methods jointly embed different KGs and put similar entities in close proximity in a vector space, as shown in Figure 4, where a nearest neighbor search can retrieve the entity alignment.

Due to its effectiveness, embedding-based entity alignment has drawn extensive attention recently. KGs have evolved to be the building blocks of many intelligent systems. They provide fundamental tools for NLP tasks [31] in language representation through BERT, knowledge reasoning [32], recommend systems using knowledge graph convolutional networks (KGCN) [33], and cross-lingual entity alignment (CEA) based on generative adversarial networks (GAN) [28] with semi-supervised learning. Despite their importance, KGs are usually costly to construct and naturally suffer from incompleteness [34]. Table 1 shows a brief survey of recent graph alignment methods on account of whether they are scalable for the IIoT domain or not. The analysis focused on the utilization of both language and structural information. It is evident that most of the models heavily rely on pre-aligned entities used during the training stage.

### 2.4. Challenges to Adaptation and Integration

For the foreseeable future, ML models will play a primary role in automating current industrial applications into intelligent solutions. However, as the previous sections highlight, research in translation among IoT devices and automatic language translation is working in isolated areas, whereas their synergy could bring greater benefits to both. The following sub-section presents the important gaps this work is based on and clear indications for plausible mergers to bridge these gaps.

#### 2.4.1. State-of-the-Art Limitations

We conducted a query search in three well-known search engines, i.e., Google Scholar, SCOPUS, and Web of Science, to investigate the existing research publications for the given problem. The main metric of this analysis was the number of publications per year.

The search queries were designed sequentially, in which the first search query was on publications for M2M translation but only within the Industry 4.0 paradigm. The second query was narrowed down to the same problem but specifically addressing ML approaches. Lastly, the third query investigated the number of publications focused on ML models for solving ontology alignment problems. Table 2 presents the statistics of search results, and the numbers indicate a lack of attention towards ML approaches for solving M2M translation problems, specifically using alignment tasks.

#### 2.4.2. Lack of NLP Fusion in the IIoT Domain

Dynamic translation between machines has stressed the need to establish automated systems that enable effective real-time communication across heterogeneous devices. The literature is unquestionably full of NLP solutions for various industrial applications, including language translation (chatbots), but most focus on a pre- or post-process analysis of processes and datasets. On the other hand, IIoT network activities are ongoing and very diverse, and there is an important need to deploy automatic translators for dynamic, seamless communication between heterogeneous devices. Using NLP models for that purpose represents a considerable gap in the available studies. As seen in Table 2, researchers place great emphasis on language datasets, even regarding graph alignment approaches. This work will be the first of many efforts to conceptualize the mapping and validate the proposed solution as a proof of concept. To understand how mapping is implemented in this study, let us dissect the NLP domain into its main components: a language encoder, a structural encoder, language sentences and tokens, and a structural question set.

#### 2.4.3. Limitations of the Dataset for IIoT Ontology Alignment

Considerable efforts have been made and will continue for the foreseeable future to develop a variety of datasets for computer-based linguistic technology applications [41]. The research community recognizes that only data can pave the way for linguistic technology. Hence, the number of publicly accessible NLP datasets has grown significantly as researchers experiment with new tasks, larger models, and novel benchmarks [42]. Datasets are essential in empirical NLP studies, since they are utilized to evaluate proposed models and for their bench-marking. Supervised datasets with predefined annotations are required to train and fine-tune models, and large unsupervised datasets are required for pre-training and language modeling. DBP15K [43], YAGO [44], and DWY100K [45] are widely used large benchmark datasets of knowledge bases for alignment tasks, with the high alignment accuracy of existing embedding-based methods. Each consists of millions of KG triplets, with thousands of entities and relations.

Whereas there is plenty of research on and datasets for cross-linguistic alignment tasks, both are scarce for industrial IoT ontology alignment. IoT ontology graphs are concise, since they are curated for specific industrial use cases and devices. As seen in Table 3, there are fewer nodes and graph triples than language dataset knowledge bases.

## 3. Problem Formulation

This section contains two key definitions designed to the address problem domain. Then, we present the problem targeted in this work.

### 3.1. Definition 1: Knowledge Graph and Structure Encoding

We generate KGs of two forerunner ontologies using W3C regulations: SSN and SOSA as KG${}_{1}$ and KG${}_{2}$. A graph is denoted as KG = (H, T, R), where R is a set of all relation entities, H is a set of all head entities, and T is a set of all tail entities. Each edge represents a relation r ϵ R, a subject node represents h ϵ H, and an object node represents t ϵ T. In the structural encoder of the proposed model, there are four representation vectors: D${}_{H}^{S}$, D${}_{R}^{S}$, D${}_{T}^{S}$, and D${}^{S}$. Vector D${}_{H}^{S}$ represents the path length from a head entity, D${}_{R}^{S}$ represents the path length from the relation, D${}_{T}^{S}$ represents the path length from a tail entity, and D${}^{S}$ encodes the structural information of the underlying KG. Entity pairs between KG${}_{1}$ and KG${}_{2}$ are denoted as

- for pair of head entities *g(h, h${}^{\prime }$)* where h ϵ H ϵ KG${}_{1}$ and h${}^{\prime }$ϵ H${}^{\prime }$ϵ KG${}_{2}$
- for pair of relation entities *g(r, r${}^{\prime }$)* where r ϵ R ϵ KG${}_{1}$ and r${}^{\prime }$ϵ R${}^{\prime }$ϵ KG${}_{2}$
- for pair of tail entities *g(t, t${}^{\prime }$)* where t ϵ T ϵ KG${}_{1}$ and t${}^{\prime }$ϵ T${}^{\prime }$ϵ KG${}_{2}$

### 3.2. Definition 2: Mapping to the BERT Language Model

The metadata, labels of nodes, and relations are conceived as the language of the IoT ontology. The language encoder of our proposed model is similar to the original BERT encoder [46]. Sets of H, R, and T along with D${}^{S}$ vectors are encoded into the B${}^{L}$ encoder, on which we apply concatenation to generate a final language representation vector, as C${}^{L}$. KG${}_{1}$ will have a matching node in KG${}_{2}$ if a node $e_{i}$ has a similar embedding vector $e_{i}^{\prime }$ in the common latent space of both KGs.

### 3.3. Problem Definition: Ontology Graph Alignment

The problem herein is manifold. Given two ontology graphs, KG${}_{1}$ and KG${}_{2}$, provided they both are designed for IIoT sensor devices, the prime task is to learn the alignment of the heterogeneous ontology graphs. For which, we first use the language BERT encoder (B${}^{L}$) on the ontology dataset and further process it using a two-layer multi-layer perceptron (MLP) network that learns the final language representation vector as C${}^{L}$. Next, we use a structural encoder to transform the language vectors into a binary vector D${}^{S}$ to capture the triplets and in-graph information with respect to neighboring nodes. Then, an interaction model is used to learn the alignment across the graphs with two baseline assumptions:

1. An entity from a KG${}_{1}$ can only match with only one entity in KG${}_{2}$. The term $C_{ij}^{uniquemax}$ ensures this property in two different KGs.
2. If an entity $e_{i}$ form KG${}_{1}$ is aligned with entity $e_{j}$ of KG${}_{2}$, then their neighbor will also have similar properties. The term $S_{i}^{topsum}$ ensures this property in the neighbor of $e_{i}$ and $e_{j}$.

Lastly, a Loss${}_{interaction}$ function is defined to learn the maximal similarity based on the side and structural information of the different entities from both KGs.

## 4. Proposed Model

### 4.1. Architecture of the Proposed System

There are two forms of information available in a KG. The first is language information, and the second is structural information. BERT-based encoders have already proven their effectiveness in language models [46]. Recently, BERT-INT, a BERT encoder, has also been used for the entity alignment task in KGs [39]. However, BERT-INT [39] only uses language information with a BERT encoder to generate an encoded vector, which is further encoded by a multi-layered-perceptron (MLP) network to yield the final representative vector for a given query.

**Figure 5:**
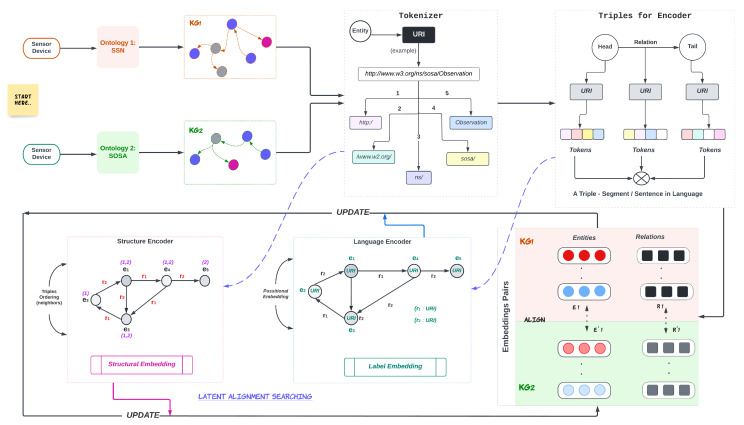
Complete overview of the proposed model with abstract components.

Indeed, the structural information is used in its interaction model in the last stage but, importantly, structural information is not covered effectively by BERT-INT. In this work, we present a model-based solution for ontology alignment using a modified BERT-INT model on graph triplets that encodes the available information in KGs, with or without pieces of language information. Figure 5 illustrates an overview of the model, starting from two heterogeneous sensor devices that have different ontologies.

### 4.2. Improvements to the BERT_INT Model

The following sections present in detail every component of the proposed model. However, here is a summary of proposed improvements to the state-of-the-art model:

- A modified input arrangement is used in this work, to utilize the full potential of a pretrained BERT model;
- The improved input arrangement can be used for experiments of aggregation models that are designed using both language and structural encoders;
- For integrating the structural encoder and incorporating side information with an improved BERT-INT model, the structural question-set reasoning block is designed and implemented with an in-graph approach;
- The interaction model is changed by proposing an iterative method of calculating similarities between entities in each iteration;
- An interaction model is designed for an unsupervised learning approach, as in the case study used for the work, where no alignment pairs are available for KG1 and KG2.

## 5. **Ontology Dataset Construction for the System Use Case**

We selected two ontologies, SOSA and SSN, as discussed in Section 2.1.3, as these are the forerunner ontologies curated by W3C on the account of IoT sensor devices. For generating ontology instances strictly on SOSA and SSN ontology graphs, we follow the W3C standardized examples of *Appartment 134* [47] and utilize the RDF (resource description framework) files containing graphs with SOSA and SSN core terms. The example is designed for temperature sensor devices and an actuator, in which the devices log their temperature values for corresponding time stamps. Although this gives us a complete graph of the ontology for sensor devices for the training of a machine learning model, we require a much larger number of ontology instances.

**Figure 6:**
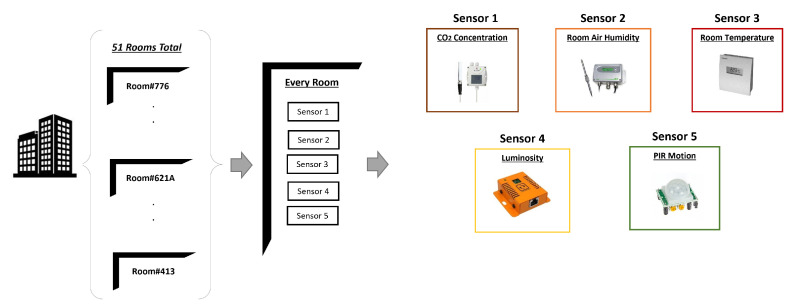
Smart building system dataset collected over a period of one week, from Friday 23 August 2013 to Saturday 31 August 2013. The PIR motion sensor was sampled once every 10 s and the remaining sensors were sampled once every 5 s. Each file contains the timestamps (in Unix Epoch Time) and actual readings from the sensor.

Therefore, we refer to Kaggle’s dataset of smart building data [48] synthesized by Hong et al. [49]. This dataset was collected from 255 sensor time series, instrumented in 51 rooms on four floors of the Sutardja Dai Hall(SDH) at UC Berkeley. This dataset can be utilized for experiments relating to IoT, sensor fusion networks, or time series tasks. It is also suitable for both supervised and unsupervised learning tasks. The building infrastructure is such that each room includes five types of measurement sensor data, as shown in Figure 6. In the following sections, we discuss the complete workflow of the proposed system for language encoding and ontology structural construction.

### 5.1. Language Encoder

**Figure 7:**
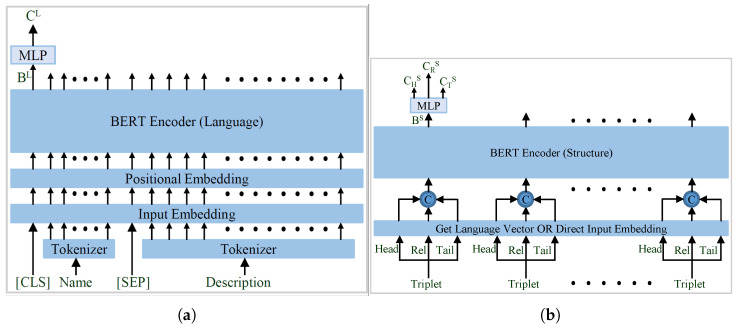
Different BERT Encoders used in the proposed model; (**a**) Encoding Structure; (**b**) Encoding Structure.

The language encoder of the proposed work is similar to BERT-INT, with modification as discussed in Section 4.2, and it generates a language representative vector for each entity and relation in the graph represented in Figure 7. Then, the language vectors of the head, relation, and tail of the triplet are concatenated to form the input vectors for the structural BERT encoder shown in Figure 7b. The corresponding embeddings generated using the structural BERT are further diverged into three separate vectors using another MLP network, to yield the final representative vector for the respective triplet’s head, relation, and tail.

**Figure 8:**
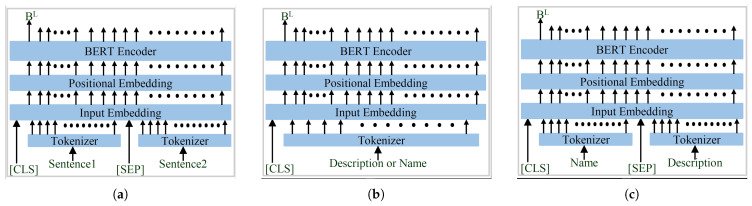
Different Input Arrangements for the BERT Encoder; (**a**) BERT; (**b**) BERT-INT; (**c**) Proposed.

The original BERT encoder [46] uses a sentence-1 and sentence-2 input arrangement, as shown in Figure 8a. The same input arrangement is utilized by most of the methods that utilize the pretrained BERT model [46]. However, BERT-INT [39] does not use this input organization and uses a very different arrangement, as shown in Figure 8b. Therefore, utilization of the full potential of a pretrained BERT model is questionable. In contrast, the input arrangement of the proposed language encoder, as represented in Figure 8c, is very similar to the original BERT encoder. Here, only the input arrangement is updated and everything else remains the same as in BERT-INT. The representation generated by the language BERT encoder (B${}^{L}$) is further processed using a two-layered MLP network, which yields the final language representation vector as $C^{L}$.

**Figure 9:**
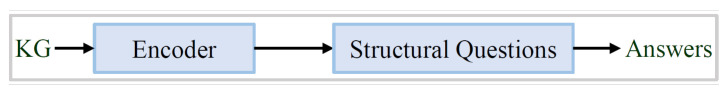
Structural Encoder Block.

The structural encoder yields an output for a given KG as input, such that the generated output can answer all questions related to the structure of the KG, as shown in Figure 9. However, there are two issues with this structural encoder:

1. How to represent the complete KG as an input?
2. What questions should be set to capture all structural information of the KG?

Processing the complete KG as input for very large KGs is not computationally feasible, so initial work tried to generate the embedding vectors for the different components of KG (such as the head (subject), relations, and tail (object)). Generating an embedding vector for a component of KG requires contextual information, but acquiring all the contextual information of a node or a relation is complex. Therefore, most existing works treated all neighbors within a specific path length as the context of the targeted node. Besides this, these embeddings should provide answers to structural questions. The most famous approaches are (1) continuous bag of words (CBOW) and (2) skip-gram for encoding structural information.

### 5.2. Structural Encoder

#### Graph Representation for Structural Encoder

In this work, we represent a graph using its set of triplets. These triplets are passed to the structural encoder to incorporate the structural information. These triplets do not have any specific order, so they are not integrated with the positional encoder. Besides this, the set of triplets passed as input at a time are considered in-graph. The components of the original graph that are not part of the in-graph are considered for structural encoder processing.Therefore, only the elements of the in-graph (nodes and relations) will participate, differentiating the entity from having different neighbors and weakening the issue of aggregating neighbors.

**Figure 10:**
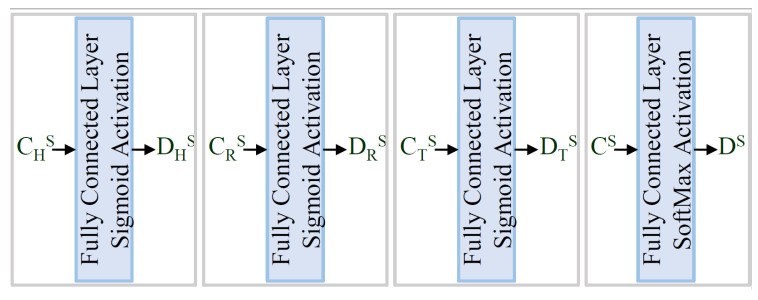
The structural question-set for encoding structural information.

We require a cost function to train the structural encoder, such that the generated representation vectors should incorporate the structural information of the underlying knowledge graph. We can ensure specific information is encoded into the representation vectors by obtaining the desired results from a linear transformation of the vector. The linear transformations shown in Figure 10 convert the representation vectors into vectors as D${}_{H}^{S}$, D${}_{R}^{S}$, D${}_{T}^{S}$, and D${}^{S}$, which represent the structural information from the knowledge graph.

The vectors generated by the structural encoder should incorporate the structural information. Therefore, a fully connected layer extracts these pieces of information from them. Figure 10 and Equation (1) explain the structural question-set used in the proposed work. Here, the vector C${}_{R}^{S}$ is transformed into a binary vector D${}_{R}^{S}$, where its *i*th element represents the connectivity of the *i*th entity with this relationship element. The vectors C${}_{H}^{S}$, C${}_{T}^{S}$ are transformed into probability vectors D${}_{H}^{S}$, D${}_{T}^{S}$, respectively, where the *i*th element represents the connectivity score of the *i*th entity with this entity. ${}^{g}$D is the reference-labeled ground truth for the corresponding vector, as shown in Equation (1).

$$\begin{array}{cc} {}_{i}^{g}D_{H}^{S} & =\left\{\begin{array}{cc} e^{-spl}, & \mathrm{spl}\;\mathrm{is}\;\mathrm{shortest}\;\mathrm{path}\;\mathrm{length}\\ \; & \mathrm{between}\;i\mathrm{th}\;\mathrm{entity}\;\mathrm{and}\;\mathrm{this}\;\mathrm{head}\\ 0, & \mathrm{no}\;\mathrm{connectivity} \end{array}\right.\\ {}_{i}^{g}D_{R}^{S} & =\left\{\begin{array}{cc} 1, & \mathrm{if}\;i\mathrm{th}\;\mathrm{entity}\;\mathrm{is}\;\mathrm{connected}\;\mathrm{with}\;\mathrm{this}\;\mathrm{relation}\\ 0, & \mathrm{otherwise} \end{array}\right.\\ {}_{i}^{g}D_{T}^{S} & =\left\{\begin{array}{cc} e^{-spl}, & \mathrm{spl}\;\mathrm{is}\;\mathrm{shortest}\;\mathrm{path}\;\mathrm{length}\\ \; & \mathrm{between}\;i\mathrm{th}\;\mathrm{entity}\;\mathrm{and}\;\mathrm{this}\;\mathrm{tail}\\ 0, & \mathrm{no}\;\mathrm{connectivity} \end{array}\right.\\ {}^{g}D^{S} & =\mathrm{one}\;\mathrm{hot}\;\mathrm{vector}\;\mathrm{for}\;\mathrm{corresponding}\;\mathrm{entity} \end{array}$$

The cost function for the learning of the parameter of the structural encoder is based on the mean square error (MRR) function. As we have multiple questions set for encoding the structural information, their corresponding losses are weighted to form the final cost (loss) of the encoder. The cost of the structural encoder ($L^{S}$) is given by Equation (1). Here, the weights s1,s2,s3 are empirically set as s1=0.3,s2=0.5,s3=0.3.

(1) $$\begin{array}{cc} \; & L_{D_{H}^{S}}=\frac{\sum_{i=0}^{n}{{(}_{i}^{g}D_{H}^{S}-iD_{H}^{S})}^{2}}{n}\\ \; & L_{D_{R}^{S}}=\frac{\sum_{i=0}^{n}{{(}_{i}^{g}D_{R}^{S}-iD_{R}^{S})}^{2}}{n}\\ \; & L_{D_{T}^{S}}=\frac{\sum_{i=0}^{n}{{(}_{i}^{g}D_{T}^{S}-iD_{T}^{S})}^{2}}{n}\\ \; & L_{D^{S}}=\frac{\sum_{i=0}^{n}{{(}_{i}^{g}D^{S}-iD^{S})}^{2}}{n} \end{array}$$

(2) $$L^{S}=s1\times L_{D_{H}^{S}}+s2\times L_{D_{R}^{S}}+s3\times L_{D_{T}^{S}}+L_{D^{S}}$$

### 5.3. Interaction Model

The proposed work utilizes two interaction model learning schemes: (1) supervised, and (2) unsupervised. The supervised interaction model learning scheme is used when we have labeled data available for training, whereas the unsupervised interaction model learning does not have any label data. These two different learning approaches use different interaction models, with some modifications.

(3) $$\begin{array}{cc} \; & S_{ij}=\frac{C_{ei}^{F}.C_{ej}^{F}}{∥C_{ei}^{F}∥.∥C_{ej}^{F}∥}\\ \; & S_{i}^{max}=\max_{j=0}^{n}\left(s_{i0},s_{i1},s_{i2},s_{in}\right) \end{array}$$

#### 5.3.1. Supervised Learning of the Interaction Model

**Figure 11:**
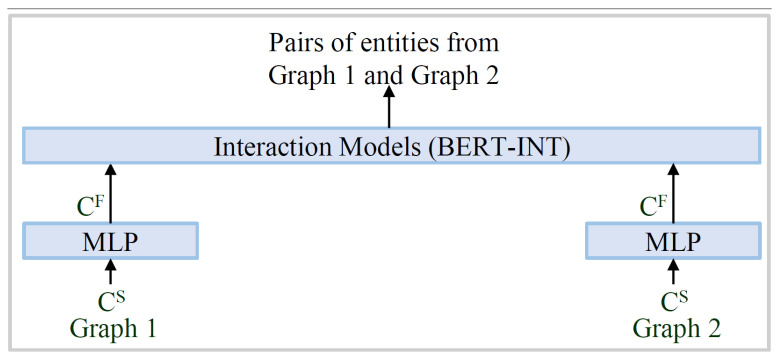
The interaction model for alignment of the entities of the different graphs.

The interaction model used in the proposed work is similar to BERT-INT (refer to Figure 11). All operations are the same, except for the calculation of the $S_{i}^{max}$ (r BERT-INT). The original BERT-INT discarded the other similarities, except the maximal one. The maximal similarity is given by Equation (3). Discarding other similarities is a waste of information, and we propose that they should be discarded after applying a softmax activation (refer to Equation (4)) across the row similarities. If we have similar entity pairs from graph 1 and graph 2, then we can maximize the corresponding $S_{ij}^{softmax}$ and then use $S_{ij}^{softmax}$ as the $S_{i}^{max}$ for the interaction model. However, if the pair information is not available (i.e., we do not have the proper pairing between the entities), then $S_{i}^{max}$ should be replaced by $S_{i}^{topsum}$, which is calculated using Equation (4). Here, N is the number of top elements (having high $S_{ij}^{softmax}$). The value of N is dynamic in nature and decreases as the learning proceeds. We decrease the value of N by one after each epoch of learning, till it becomes one.

(4) $$\begin{array}{cc} \; & S_{ij}=\frac{C_{ei}^{F}.C_{ej}^{F}}{∥C_{ei}^{F}∥.∥C_{ej}^{F}∥}\\ \; & S_{ij}^{softmax}=\frac{e^{\alpha^{2}s_{ij}}}{\sum_{j=0}^{n}e^{\alpha^{2}s_{ij}}}\\ \; & S_{i}^{TopN}=\left\{S_{ij}^{softmax}|S_{ij}^{softmax}\in \mathrm{top}\;N\;\mathrm{elements}\;\mathrm{of}\;S_{i}\;\mathrm{row}\right\}\\ \; & S_{i}^{topsum}=\sum_{S_{ij}^{softmax}\in S_{i}^{TopN}}\left(S_{ij}^{softmax}\right)\\ \; & Loss_{interaction}=\mathrm{same}\;\mathrm{as}\;\mathrm{BERT-INT} \end{array}$$

#### 5.3.2. Unsupervised Learning of the Interaction Model

The interaction model used for this scheme is different from BERT-INT. Here, we do not have pair alignment information for the entities of KG 1 and KG 2. Therefore, we need to reduce the trainable parameter of the interaction model, as there is no validated gradient (corresponding to the ground truth label) for parameter learning. The proposed work also does not utilize the dual-aggregation technique for unsupervised learning, as we do not want to use a trainable MLP for the final classification. This new interaction model is defined by Equation (5). Here, as we do not have any alignment information available, we need to utilize only the implicit information of the different entities from different KGs. The two properties (assumption) we are exploiting for the learning are mentioned in Section 3.3.

(5) $$\begin{array}{cc} \; & Dist_{ij}=C_{i}^{F}-C_{j}^{F}\\ \; & C_{ij}^{uniquemax}=\frac{e^{\beta^{2}Dist_{ij}}}{\sum_{i=0}^{m}e^{\beta^{2}Dist_{ij}}+\sum_{j=0}^{n}e^{\beta^{2}Dist_{ij}}-e^{\beta^{2}Dist_{ij}}}\\ \; & S_{ij}=C_{ei,ej}^{uniquemax}\\ \; & S_{i}^{TopN}=\left\{S_{ij}|S_{ij}\in \mathrm{top}\;N\;\mathrm{elements}\;\mathrm{of}\;S_{i}\;\mathrm{row}\right\}\\ \; & S_{i}^{topsum}=\sum_{S_{ij}\in S_{i}^{TopN}}\left(S_{ij}\right)\\ \; & Loss_{interaction}=\sum_{All}\left(1.0-S_{i}^{topsum}\right) \end{array}$$

## 6. Experimental Setup

### 6.1. Training Procedure

In this section, we elaborate on the training procedure used for the experiments. We utilized the Adam optimizer to train the proposed system with a dynamic learning (exponentially and linear decreasing) rate setting. The learning rate was initialized to 0.001 and reduced to $10^{-4}$ in 25 thousand iterations, with an exponentially decaying rate. After 25 thousand iterations, we operated a linearly decaying learning rate, as in Equation (6). A total of one million iterations with 16 batch sizes were used to train the proposed system. The learning stage also included L2 regularization with a scale of $10^{-4}$, to limit overfitting in the trained system.

(6) $$lr=10^{-4}\times \left(1.01-\frac{iterationCount}{2,500,000}\right)$$

### 6.2. Evaluation Metric

Consistently with the previous works in the literature, Hits@k (k = 1, and 10) and mean reciprocal rank (MRR) were selected as the evaluation metrics in this paper. Hits@k calculates the proportion of correctly aligned entities ranked in the top-k list. Here, we focused on Hits@1 and Hits@10. MRR measures the average of the reciprocal ranks of the results. Outstanding methods should have a higher Hits@k and MRR. Furthermore, during training, a 30–70% split of the dataset was applied by consciously taking out the data of floor#4 to be used during validation.

### 6.3. Experiments Breakout

**Figure 12:**
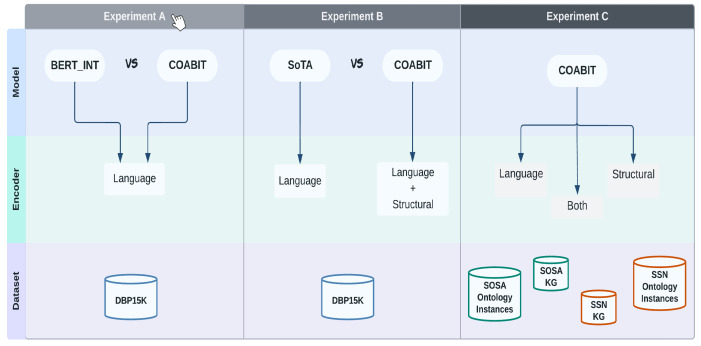
Layout of the experiments and the components used in them.

The empirical study for this work was designed with three different experiments, as shown in Figure 12.

Experiments were designed from a systematically logical perspective. First, we conducted a comparative analysis of the baseline model with our proposed model. Next, we evaluated the performance of the proposed model in contrast to the state-of-the-art methods. Lastly, we conducted an ablation study on the proposed model, to study the architecture’s effectiveness. The selection of datasets in each set of experiments is also mentioned in Figure 12.

## 7. Proof of Concept and Results

### 7.1. Improvement on SoTA (BERT_INT vs. Proposed)

As discussed earlier, the proposed model was designed as a similar model to BERT-INT but with the modifications explained in Section 4.2 and Section 5.3. We extended the experiments of language encoder-based graph alignment conducted by Tang et al. [39] by using the same DBP15K dataset and similar BERT embedding setting and evaluated the results using the same parameters of HitRatio@K (K = 1, 10) and MRR. The modification of the language encoder involved updating the input arrangement shown in Figure 8b. The effectiveness of this input arrangement was also verified by incorporating it within BERT-INT, as shown in Table 4. The table’s first row shows that the BERT-INT model’s performance improved when the proposed input arrangement was used. The second row shows the results of the proposed model only using the proposed language encoder with the modified input arrangement. The results clearly show that even minor improvements bettered the BERT-INT model. Moreover, we compared the complete proposed model (language + structural encoder) with the state-of-the-art results presented in [39] in Table 5, and it can be seen that the performance of the proposed model was the highest, by approximately 1.2–2.7%.

### 7.2. Quantitative Analysis with Ablation Study

To thoroughly investigate the effectiveness of the proposed encoders, we conducted an ablation study on the proposed model. The dataset used for these experiments was the synthesized ontology dataset created from the smart building dataset of Kaggle, as discussed in Section 5, using SOSA and SSN ontology graphs. In Table 6, the first set of experiments were on *Synthetic SOSA—KG SSN*, in which the MMR score was highest when both encoders were used. For experiments with only the KG structure, the interaction model was pretrained on a known ontology and used the direct input embedding vector for the corresponding entity. However, the MRR score was lowest when only the structural encoder was used, which indicates that enforcing the graph structural information might have excluded all those alignment matches that were correct with respect to the language encoder but incorrect as per the ontology. A similar pattern was observed in the other experiment sets as well. The last key observation is that the highest HRs and MRR scores were achieved when the *KG SOSA—Synthetic SSN* dataset was used. Our reflection on this is that SSN is a superset of SOSA, so the model might have found all the correct alignments for every token of SOSA. Additionally, all alignment results had to be validated using annotations hand-picked by a human expert, as no bench-marking ontology alignment dataset was present. Although these results are subjective to the alignment annotations, they are important because of their novelty.

### 7.3. Qualitative Analysis of the Proposed Model

**Figure 13:**
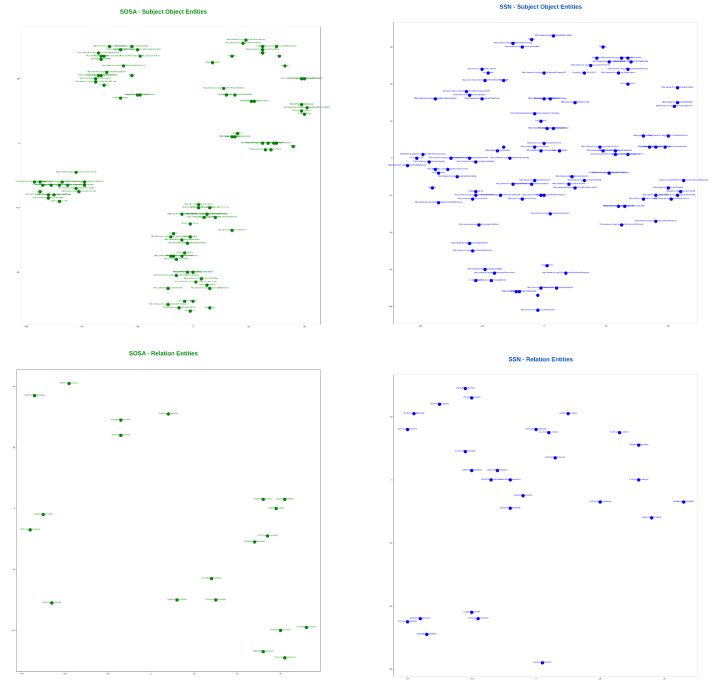
tsne plots generated from vectors of SOSA and SSN entities. An entity can be a node (subject or object) or a relation.

**Figure 14:**
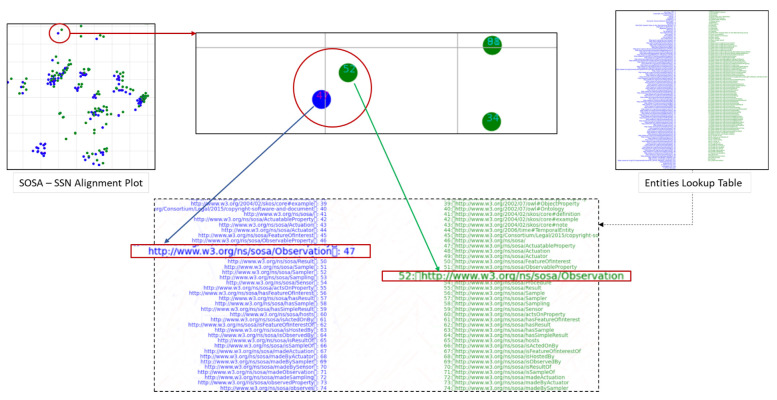
Ontology graph alignment pair demonstration. Entities in blue represent SOSA graph nodes and green represent SSN graph nodes. For clarity and ease of visualization, all SSN nodes in the alignment plot are shifted three spaces to the left.

**Figure 15:**
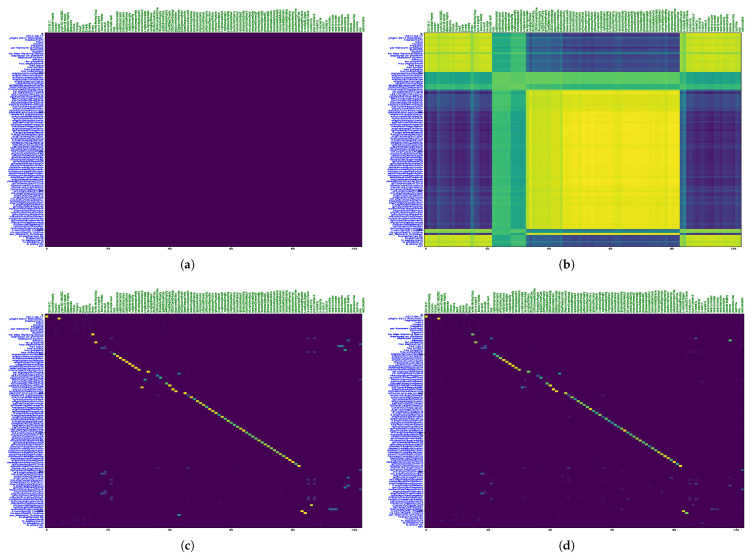
DistMap between Different Nodes of SOSA and SSN KGs; Sub-Sub DistMap at (**a**) 1000 iteration; (**b**) 18,000 iteration; (**c**) 35,000 iteration; (**d**) 62,000 iteration.

**Figure 16:**
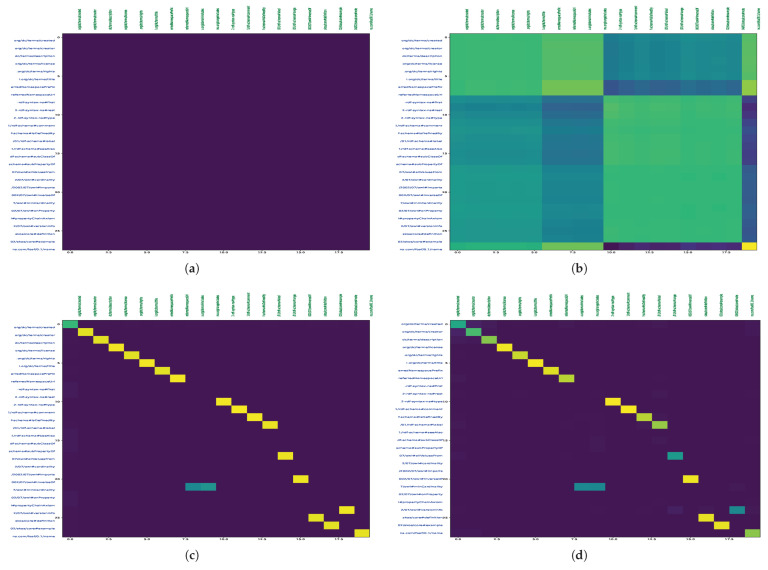
DistMap between Different Relations of SOSA and SSN KGs; Rel-Rel DistMap at (**a**) 1000 iteration; (**b**) 18,000 iteration; (**c**) 35,000 iteration; (**d**) 62,000 iteration.

To visualize the alignments, we generated tsne plots of all the entities from both ontologies. First, we performed indexing of all nodes and relations for both SOSA and SSN ontologies. Then, lookup tables for the entities were created. Next, we reduced the embedding vectors of all entities into two-dimensional tsne plots, as shown in Figure 13. Figure 14 demonstrates an alignment pair. Here, we magnified a pair of adjacent nodes from the alignment plot and followed their index in the lookup tables. We can see that both nodes were similar across the ontology; hence, they were aligned in the plot with the smallest Euclidean distance. Additionally, for further analysis of all entities, the tsne plots were used to create heat-maps by calculating the Euclidean distance maps shown in Figure 15 and Figure 16. These figures also show the learning of the model throughout iterations from 1000 to 62,000th iteration. The heat maps show the one-to-one mapping between pairs of SOSA and SSN nodes and relations, respectively. In the beginning, the model learned almost no mapping, but the processing of loss functions continued; it started identifying similar entities and those with lesser Euclidean distances between them are highlighted with lighter colors on the map.

## 8. Conclusions and Future Work

This paper is the first to conceptualize ontology alignment for the Industrial Internet of Things (IIoT) domain based on a natural language processing (NLP) model for alignment among heterogeneous devices. The proposed model characterizes the ontological meta-data as side information and the structure as the schema and learns vector embeddings for all entities and relations. In extensive experiments on both cross-lingual and cross-ontology tasks, our model consistently outperformed the baseline BERT_INT model by 1.2–2.7% in HR and MRR scores. However, these results have a few pertinent limitations. First, the ontology dataset had to be synthesized, due to the lack of publicly available real-world smart sensor datasets. While language translation undoubtedly has a solid foundation and large datasets are available for human language ontology, this is not true for the IIoT domain. Second, there is no bench-marking dataset available for establishing the ground truth for IoT ontology alignment; therefore, the alignments between SSN and SOSA ontology were annotated by human experts. Although the results may be subjective due to the alignment annotations, they are important because of their novelty. Last, the ontology graphs of IoT ontology for sensor devices are very concise by design. The number of unique entities (nodes + relations) and triples in them is maximum in the hundreds, as opposed to language ontology, which usually has thousands of nodes. For instance, the SSN ontology has 125 unique entities, while SOSA has 75, so the accuracy results of correct alignments in Table 6 are as per the limited number of unique entities. Moreover, the ontologies for sensor devices were designed for functionally similar types of devices but with varying design principles. Nevertheless, when the model learns language embeddings, it is easier to find nodes across ontologies that have labels with similar semantic meanings. To remove any such biases, a structure encoder was utilized to impose the context by correctly aligning only those nodes with matching labels and similar in-graphs (neighbors).

There are several directions this work could potentially develop in. A generalized IoT ontology designed for any IoT device (beyond sensors) could be tested for ontology alignment, to make an even stronger ablation study. One such ontology is SAREF [50], which has approximately 1097 unique triples, the maximum among any IoT ontology. Another potential future work is that the paucity of benchmarking datasets could be resolved by conducting crowd-sourcing of a ground truth to build validation data for IoT ontology alignment and annotations. There are public platforms such as BioPortal being used for medical research that provide annotations for disparate biomedical ontologies [51]. Inspired by this, IoT ontological resources could also be publicly provided for research, to remove the bottlenecks of dataset limitations. Last but not least, as this work can be considered a step towards enabling translation between heterogeneous IoT sensor devices, the proposed model could be extended to a translation module in which, based on the ontology graphs of any device, the model could interpret the messages transmitted from that device. This idea is at an abstract level as of now and needs extensive efforts and empirical studies to be realized fully.

## Figures and Tables

**Table 1:** Summarizing recent and renowned state-of-the-art methods for the graph alignment task.

Ref.	Learning Approach	Entity Matching	Domain	Datasets Used	Structure Information Used?	Scalable for IIoT Domain?
[35]	supervised	MTransE based	Languages	WK31-15k	✓	yes, but only if benchmark datasets is present
[36]	supervised	GCN based entity embeddings	Languages	DBP15K	✓	yes, but only if all pre-aligned entities are included in training data
[37]	supervised	Topic entity graph using GCN	Languages	DBP15K	✗	no, as each entity in ontology graph represents a topic and topic grouping will fail here
[38]	unsupervised	TransE based predicate alignment for attribute character embeddings	Locations	DBP, GEO, YAGO	✓	no, as structure information is used only to learn the relations labels and not the interconnections
[39]	supervised	BERT based Interaction Model	Languages	DBP15K	✗	yes, but only with unsupervised learning approach and inclusion of structure information
[40]	semi-supervised	BERT for Triadic KG	Languages	DBP15K	✗	no, as ontology graphs can not be realized as triadic KG with all independent entities

**Table 2:** Details of the search queries in different search engines and search results in the number of publications in every year. Searching in Google Scholar was on “Entire Article”, and SCOPUS was on “Title, Abstract, Keywords”, while Web of Science was only on “Abstract”.

Year	Query 1: M2M Translation & Industry 4.0			Query 2: M2M Translation & Industry 4.0 & ML			Query 3: M2M Translation & Industry 4.0 & ML & Ontology Alignment		
	Google Scholar	SCOPUS	Web of Science	Google Scholar	SCOPUS	Web of Science	Google Scholar	SCOPUS	Web of Science
**2010**	10,200	95	76	9180	0	0	232	0	0
**2011**	11,600	89	85	10,900	0	0	229	0	0
**2012**	12,900	101	94	12,800	0	0	231	0	0
**2013**	13,400	116	93	14,500	0	0	233	0	0
**2014**	15,000	220	140	16,100	0	2	202	0	1
**2015**	16,000	344	306	17,100	2	3	196	0	1
**2016**	18,500	751	500	17,500	21	8	211	0	0
**2017**	16,600	1432	957	16,600	49	24	201	1	0
**2018**	22,900	2495	1501	19,600	146	77	206	1	1
**2019**	16,800	4886	2242	16,700	329	128	210	1	1
**2020**	29,700	5577	2514	22,900	496	148	236	1	1
**2021**	37,300	6706	3118	26,700	791	225	143	1	0
**2022**	45,120	8954	3118	22,950	977	225	298	2	0

**Table 3:** Statistics of the empirical NLP datasets used for entity alignment in two domains: (a) contemporary language-based and (b) IIoT domain utilizing both structure and language-based alignments.

Dataset		Entities	Relations	Triples
**Domain: Language-based**				
DBP15K${}_{ZH-EN}$	Chinese	66,469	2830	153,929
	English	98,125	2317	237,674
DBP15K${}_{JA-EN}$	Japenese	65,744	2043	164,373
	English	95,680	2096	233,319
DBP15K${}_{FR-EN}$	English	66,858	1379	192,191
	French	105,889	2209	278,590
**Domain: IIoT Language + Structure-based**				
Smart Appliance REFerence (SAREF)		37	20	1097
Semantic Actuator Network (SAN)		17	17	271
Semantic Sensor Network (SSN)		105	40	767
Sensor, Observation, Sample, & Actuator (SOSA)		70	23	487

**Table 4:** Experiment A, results of the performance of supervised entity alignment using the BERT_INT method and its variant with the proposed input arrangement on DBP15K dataset.

Method	DBP15K${}_{\mathrm{ZH}-\mathrm{EN}}$			DBP15K${}_{\mathrm{JA}-\mathrm{EN}}$			DBP15K${}_{\mathrm{FR}-\mathrm{EN}}$		
	HR1	HR10	MRR	HR1	HR10	MRR	HR1	HR10	MRR
BERT-INT	96.8	99.0	97.7	96.4	99.1	97.5	99.2	99.8	99.5
**Proposed**	**97.1**	**99.1**	**97.9**	**96.9**	**99.1**	**97.9**	**99.3**	**99.8**	**99.6**

**Table 5:** Experiment B, results of the overall performance of graph alignment on the DBP15K dataset using SoTA and the proposed models.

Method	DBP15K${}_{\mathrm{ZH}-\mathrm{EN}}$			DBP15K${}_{\mathrm{JA}-\mathrm{EN}}$			DBP15K${}_{\mathrm{FR}-\mathrm{EN}}$		
	HR1	HR10	MRR	HR1	HR10	MRR	HR1	HR10	MRR
Only use graph structures by variant TransE									
MTransE	30.8	61.4	36.4	27.9	57.5	34.9	24.4	55.6	33.5
IPTransE	40.6	73.5	51.6	36.7	69.3	47.4	33.3	68.5	45.1
BootEA	62.9	84.8	70.3	62.2	85.4	70.1	65.3	87.4	73.1
RSNs	50.8	74.5	59.1	50.7	73.7	59.0	51.6	76.8	60.5
TransEdge	73.5	91.9	80.1	71.9	93.2	79.5	71.0	94.1	**79.6**
MRPEA	68.1	86.7	74.8	65.5	85.9	72.7	67.7	89.0	75.5
Only use graph structures by variant TransE plus GCN									
MuGNN	49.4	84.4	61.1	50.1	85.7	62.1	49.5	87.0	62.1
NAEA	65.0	86.7	72.0	64.1	87.3	71.8	67.3	89.4	**75.2**
KECG	47.8	83.5	59.8	49.0	84.4	61.0	48.6	85.1	61.0
AliNet	53.9	82.6	62.8	54.9	83.1	64.5	55.2	85.2	65.7
Only use graph structures by variant TransE plus adversarial learning									
AKE	32.5	70.3	44.9	25.9	66.3	39.0	28.7	68.1	41.6
SEA	42.4	79.6	54.8	38.5	78.3	51.8	40.0	79.7	**53.3**
Combine graph structures and side information by variant GCN									
GCN-Align	41.3	74.4	54.9	39.9	74.5	54.6	37.3	74.5	53.2
GM-Align	67.9	78.5	-	74.0	87.2	-	89.4	95.2	-
RDGCN	70.8	84.6	74.6	76.7	89.5	81.2	88.6	95.7	91.1
HGCN	72.0	85.7	76.8	76.6	89.7	81.3	89.2	96.1	**91.7**
DGMC	77.2	89.7	-	77.4	90.7	-	89.1	96.7	-
Combine graph structures and side information by multi-view learning									
JAPE	41.2	74.5	49.0	36.3	68.5	47.6	32.4	66.7	43.0
MultiKE	50.9	57.6	53.2	39.3	48.9	42.6	63.9	71.2	66.5
JarKA	70.6	87.8	76.6	64.6	85.5	70.8	70.4	88.8	76.8
HMAN	87.1	98.7	-	93.5	99.4	-	97.3	99.8	-
CEAFF	79.5	-	-	86.0	-	-	96.4	-	-
BERT_INT	96.8	99.0	97.7	96.4	99.1	97.5	99.2	99.8	**99.5**
Graph structural encoder in conjunction with language encoder									
**Proposed**	**98.1**	**99.2**	**98.3**	**97.2**	**99.2**	**98.1**	**99.4**	**99.8**	**99.6**

**Table 6:** Experiment C, results of the ablation study using the proposed ontology alignment model on a smart building dataset with an unsupervised learning approach.

Model Used		Result in Percentage		
Language Encoder (Side Information)	Structural Encoder (KG Structure)	HR@1	HR@10	MRR
*Synthetic SOSA—KG SSN*				
✓	✓	87.6	94.3	89.5
✗	✓	81.9	88.6	**82.8**
✓	✗	83.8	92.4	**84.8**
*KG SOSA—KG SSN*				
✓	✓	80.9	91.4	83.8
✗	✓	75.2	88.6	77.1
✓	✗	77.1	90.5	79.0
*KG SOSA—Synthetic SSN*				
✓	✓	88.4	94.7	**90.3**
✗	✓	**70.1**	**76.9**	**73.2**
✓	✗	82.5	93.2	84.3

## Data Availability

Not applicable.
